# Thoracolumbar fascia thickness and body mass index as predictors of pain sensitivity: a single-blinded experimental hypertonic saline study

**DOI:** 10.1186/s12891-026-09992-7

**Published:** 2026-05-22

**Authors:** Philipp Axmann, Carla Jung, Ali Darwich, Aditya Vadgaonkar, Franz-Joseph Dally, Steffen Schulz, Alexander Blümke, Frederic Bludau, Sascha Gravius, Andreas Schilder

**Affiliations:** Department of Orthopaedic and Trauma Surgery, Medical Faculty Mannheim, University Medical Centre Mannheim, University of Heidelberg, Theodor-Kutzer-Ufer 1-3, 68167 Mannheim, Germany

**Keywords:** Thoracolumbar fascia, Fascia thickness, Nociceptive sensitivity, Body mass index, Hypertonic saline injection, Ultrasound imaging

## Abstract

**Background:**

The thoracolumbar fascia (TLF) is a key connective tissue structure that is involved in biomechanics of the lumbar spine and nociceptive processing. Growing evidence indicates that body mass index (BMI) is associated with connective-tissue remodeling, that may influence pain sensitivity and may thus contribute to musculoskeletal pain. However, the extent to which BMI-related differences in fascia morphology predict pain responses under controlled experimental conditions remains insufficiently understood. This study investigated thoracolumbar fascia thickness and BMI as predictors of pain sensitivity using a single-blinded experimental hypertonic saline pain model.

**Methods:**

Twenty healthy adult volunteers (mean age 23.6 ± 2.4 years) received injections of hypertonic saline (5.8% NaCl) into the TLF to evoke low back pain. Induced Pain intensity was assessed using numerical rating scales (0–100 NRS) and the TLF thickness was measured via ultrasound at injection and contralateral site by a person blinded to pain ratings.

**Results:**

TLF thickness was a significant predictor of experimentally induced pain sensitivity, demonstrating a moderate positive correlation with the individual peak pain (Pearson’s *r* = 0.606, *P* < 0.01), accounting for approximately 37% of the variance in pain response. Furthermore, BMI was positively associated with TLF thickness (*r* = 0.495, *P* < 0.05), indicating that higher body mass predicts increased fascial thickness. Notably, BMI was positively correlated with induced pain intensity (*r* = 0.565, R^2^ = 0.320, *P* < 0.05), supports the notion that body composition may contribute to inter-individual variability in pain sensitivity. No significant side-to-side differences in TLF thickness were observed.

**Conclusion:**

These findings identify both thoracolumbar fascia thickness and BMI as potential predictors of low back pain sensitivity and demonstrate significant associations between structural fascial characteristics, body composition, and nociceptive responses. The results are consistent with clinical observations linking higher BMI to increased musculoskeletal pain burden and suggest that fascial structural characteristics may be related to nociceptive sensitivity. Furthermore, ultrasound-based assessment of fascial thickness may represent a clinically accessible parameter associated with pain sensitivity and may be useful for characterizing musculoskeletal outcomes in the context of weight-related interventions.

## Background

Fascia thickness has emerged as a significant factor in understanding pain sensitivity across a range of musculoskeletal conditions. In plantar fasciitis, individuals with symptomatic limbs exhibit markedly thicker plantar fascia than asymptomatic controls, and the thickness of the connective tissue correlates positively with the ongoing intensity of pain [27]. Similarly, age‑related increases in fascial thickness have been linked to reductions in joint flexibility and the development of chronic pain [28]. Furthermore, eccentric exercise represents another context in which fascia dynamically remodels in association with pain. Following high‑load eccentric contractions, connective tissue thickness increases by approximately 17% within 48 h, mirroring the time course of delayed‑onset muscle soreness [21], which has been proposed to indicate a sensitization of fascia-afferent pathways [2, 3].

Despite these associations, thicker fascia layers do not invariably predict increased pain sensitivity. Interindividual differences in muscle tone, nociceptive thresholds, and central pain modulation may modulate the relationship between fascial morphology and perceived pain. For instance, in hypermobile Ehlers–Danlos syndrome, elevated fascial thickness coexists with variable pain experiences attributable in part to altered muscle stiffness and proprioceptive deficits [26] as the fascial network plays a crucial role in pain perception [16] and musculoskeletal function.

The thoracolumbar fascia (TLF) has gained particular interest as a structural and nociceptive link to low back pain. Composed of dense collagen fiber networks interspersed with adipose tissue and innervated by free nerve endings [5, 7], the TLF contributes to lumbar spine stability and responds adaptively to mechanical loading and posture changes [4]. Histological and imaging studies in chronic low back pain (CLBP) patients have documented TLF thickening suggesting loss of anisotropy or fascial remodeling [29, 15] that might reflect and sustain nociceptive sensitization.

Experimentally, hypertonic saline injection or electrical stimuli applied to deep tissues offer controlled models for probing fascia‑mediated pain mechanisms [16, 17, 25]. However, the substantial interindividual variability in pain responses indicates that the anatomical and biomechanical substrates underlying nociceptive sensitivity are not yet fully understood. Obesity represents one such potential substrate, with human imaging studies demonstrating a positive association between elevated body mass index (BMI) and increased fascial thickness. [28].

To date, the extent to which baseline TLF morphology predicts sensitivity to noxious chemical stimulation and how anthropometric measures such as BMI modulate this relationship has not yet been systematically examined. Accordingly, the present study quantified ultrasound‑derived TLF thickness in twenty healthy volunteers and assessed its association with peak pain intensity elicited by 5.8% NaCl injection. We also examined the relationship between TLF thickness and BMI, and evaluated side‑to‑side symmetry of fascial thickness to validate the contralateral site as an internal control. By elucidating structural correlates of nociceptive sensitivity and their relation to body composition, this work aims to advance our understanding of fascia role in early pain processing and to develop preventive and therapeutic strategies targeting fascial tissues.

## Material and methods

### Participants

A total of twenty healthy adult volunteers (23.6 ± 2.4 years, mean ± SD; 10 female/10 male) participated in this study. All participants were free of acute or chronic pain conditions and had no prior history of low back pain or musculoskeletal disorders. Written informed consent was obtained from all participants. The local Ethics Committee of the Medical Faculty Mannheim, University Heidelberg approved the experimental protocol on human volunteers (2023–504) according to the current version of the Declaration of Helsinki. Clinical trial number: not applicable.

Ultrasound Assessment of Thoracolumbar Fascia Thickness.

**Fig. 1:**
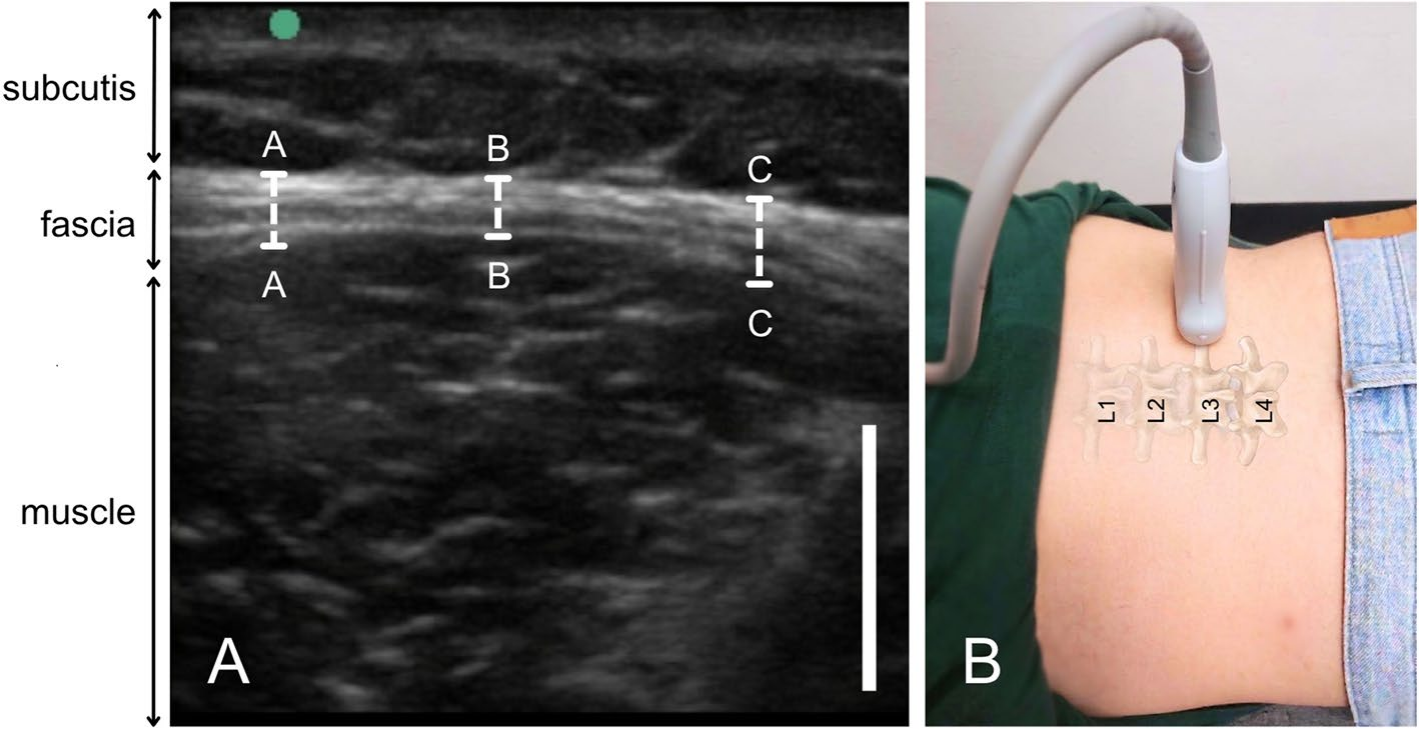
Ultrasound measurement of the thoracolumbar fascia. **A** Ultrasound image illustrating the relevant tissue layers (subcutis, fascia, and muscle). The ultrasound image was taken at the L3/L4-level. Fascia thickness was determined by averaging measurements taken at three exemplary points (A, B, and C). **B** Experimental setup demonstrating the participant in a prone position with relaxed back musculature. To ensure a constant application pressure across all subjects, the ultrasound transducer was placed on the skin supported solely by its own weight without additional manual compression

Thoracolumbar fascia (TLF) thickness was assessed at lumbar level L3/L4 using high-resolution ultrasound imaging (M-Turbo ultrasound system; Sonosite, Munich, Germany). As illustrated in Fig. 1, ultrasound imaging of the injection site was performed in transversal axis of the probe with the participant in a prone position and relaxed lumbar posture. Fascia thickness was then measured at three independent locations within the same image frame to account for regional variation (Fig. 1A). Noteworthy, determining the thickness of the fascia was conducted by an additional investigator that was blinded to the participants' pain ratings in order to reduce observational bias. Both investigators underwent structured training in musculoskeletal ultrasound imaging, including specific instruction in thoracolumbar fascia assessment. Thickness measurements refer primarily to the hyperechoic posterior layer of the thoracolumbar fascia, excluding adjacent muscle and subcutaneous tissue. Then, the average of these three values was calculated as mean and used as the representative thickness for that location and side. Consistent transducer pressure and orientation during acquisition were ensured by using the intrinsic weight of the transducer (Fig. 1B), without additional manual compression, thereby avoiding deformation of the underlying tissue. As determination of fascia thickness was not repeated at identical locations, formal intra-rater reliability analysis was not applicable.

### Pain induction and rating

To induce localized deep tissue pain, 200 µL of 5.8% hypertonic saline (NaCl) was injected into the TLF on one side of the lower back (L3/L4) as described earlier [16, 25]. Pain intensity was recorded using a 0–100 numerical rating scale (NRS), a widely used and validated measure of pain intensity [8], with 0 representing “no pain” and 100 representing “worst imaginable pain”. Participants were asked to rate chemically induced pain every 20 s following injection, and the highest reported value was defined and noted as the peak pain intensity. Based on prior evidence from similar models suggesting no secondary increase in pain, pain intensity ratings were collected until peak pain was reached and discontinued thereafter [25, 16]. All NRS assessments were conducted by an investigator blinded to the ultrasound results.

### Body mass index

Body mass index (BMI) was calculated for each participant using the standard formula: BMI = weight (kg)/height (m^2^), obtained via self-report during the initial anamnesis interview. These values were used to examine the relationship between individual body composition and TLF morphology.

### Statistical analysis

All statistical analyses were performed using GraphPad Prism (version 10.5.0, GraphPad Software, San Diego, CA). Pearson correlation coefficients (r) were used to assess the linear relationships between TLF thickness and peak pain intensity, where the thickness of the tissue underlying the injection site was used for the analyses. Pearson correlation coefficients (r) were further used to assess the linear relationships between TLF thickness and BMI, where the thickness of both, the injection site and control site, has been used as mean for the analyses. In addition, partial correlation analyses controlling for BMI were conducted to evaluate whether the association between thoracolumbar fascia thickness and pain intensity remained independent of body mass index. Correlation coefficients (r), coefficients of determination (R^2^), and corresponding 95% confidence intervals were calculated. The strength of the correlations was evaluated based on the magnitude of r (0.0–0.3 are considered negligible, 0.3–0.5 low, 0.5–0.7 moderate, 0.7–0.9 high and > 0.9 very high [12]. Paired t-tests were applied to compare TLF thickness between the injection and control sites. Intraclass correlation coefficients (ICC) were calculated to determine the reliability of side-to-side measurements. A significance threshold of α = 0.05 was applied. This study was conducted as an exploratory, hypothesis-generating investigation. A convenience sample of twenty healthy volunteers was included due to limited prior data on the association between thoracolumbar fascia thickness, BMI and experimentally induced pain sensitivity.

## Results

**Fig. 2:**
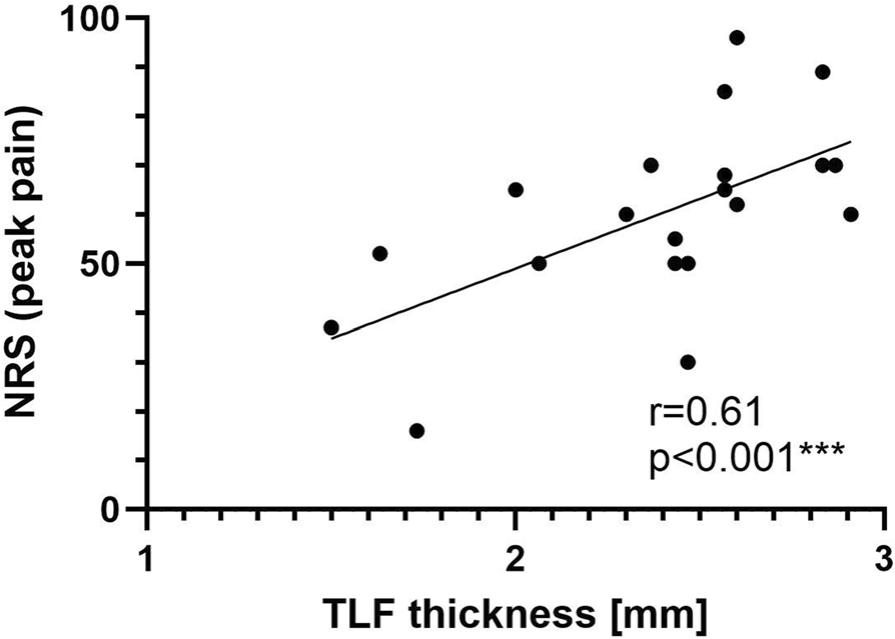
Scatter plot of the correlation between injection induced peak pain intensity on a numerical rating scale (0–100 NRS) and the thoracolumbar fascia (TLF) thickness (mm) at the injection site. (*n* = 20)

In a cohort of twenty healthy adult volunteers (23.6 ± 2.4 years, mean ± SD), the correlation between ultrasound‐derived thoracolumbar fascia (TLF) thickness and the maximal pain intensity (0–100 NRS) reported during injection of 5.8% NaCl into the TLF was examined (Fig. 2). The analysis revealed a moderate positive correlation (*r* = 0.6057, Crude Pearson correlation), indicating that approximately 37% of the variance in reported peak pain intensity could be explained by fascial thickness (R^2^ = 0.3669). The 95% confidence interval for the correlation coefficient ranged from 0.2230 to 0.8267. To account for the potential influence of body mass index, an additional partial correlation analysis controlling for BMI was performed. The association between thoracolumbar fascia thickness and pain intensity remained statistically significant after adjustment for BMI (*r* = 0.5240, *p* = 0.018*; Adjusted (BMI-controlled) partial correlation), although with a reduced correlation strength compared to the crude analysis.

**Fig. 3:**
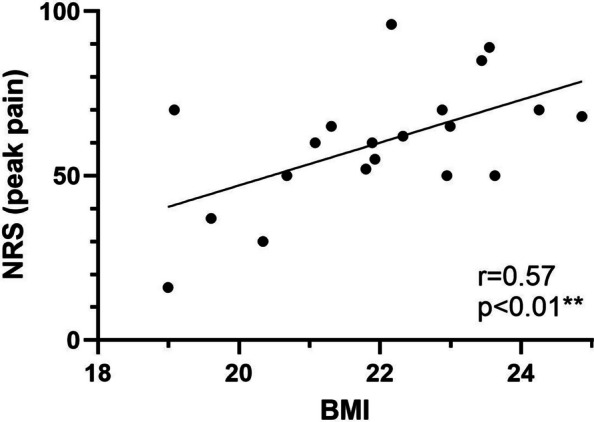
Scatter plot of the correlation between injection induced peak pain intensity on a numerical rating scale (0–100 NRS) and body–mass index (kg/m.^2^; BMI). (*n* = 20)

Furthermore, statistical analyses revealed a positive correlation (r = 0.5653), with 32% of the variation in BMI attributable to induced pain intensity (R^2^ = 0.3196). The 95% confidence interval for the correlation coefficient ranged from 0.1638 to 0.8062 (Fig. 3). The r-values presented in Figs. 2 and 3 represent crude Pearson correlation coefficients.

**Fig. 4:**
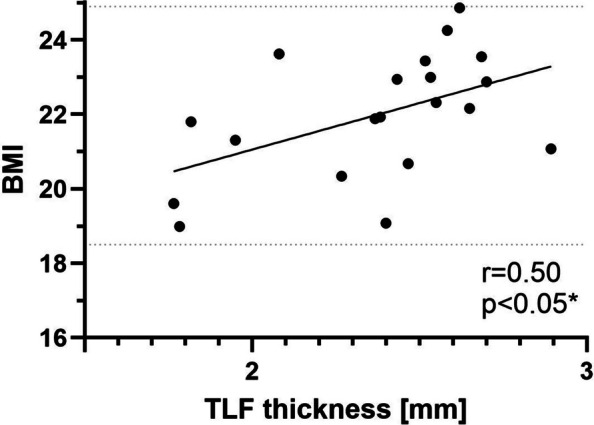
Scatter plot of the correlation between body–mass index (kg/m.^2^; BMI) and the thoracolumbar fascia (TLF) thickness (mm) of both body sites. The “normal” BMI range (18.5—24.9 BMI) is indicated between two horizontal dotted lines. (n = 20)

This study further explored the association between the measured TLF thickness and the calculated body mass index (BMI). BMI was calculated for each participant and varied in the study cohort in weight (69.2 ± 11.2 kg) and height (176.9 ± 11.0 cm; mean ± SD). Analyses revealed a moderate positive association (*r* = 0.4952), with 25% of the variation in fascial thickness attributable to differences in BMI (R^2^ = 0.2452). The 95% confidence interval for the correlation coefficient ranged from 0.0674 to 0.7692 (Fig. 4).

**Fig. 5:**
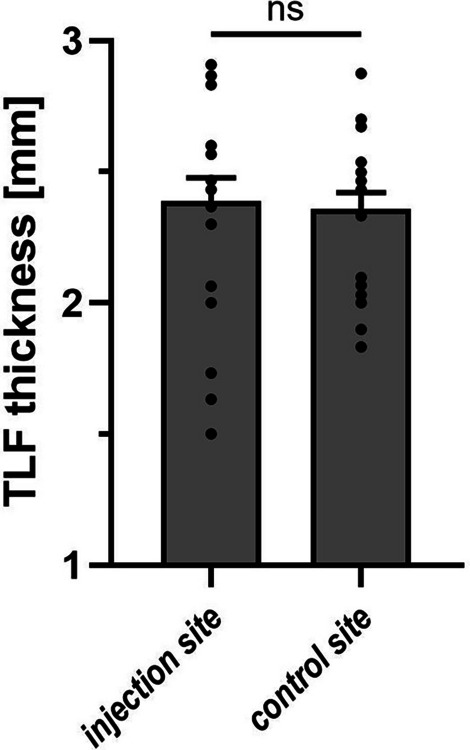
Comparison of thoracolumbar fascia (TLF) thickness (mm) at the saline injection site versus the contralateral control site. (mean ± SEM; *n* = 20)

To determine whether fascial thickness differed between the saline injection site and the contralateral control site, measurements were compared within subjects. No significant side‐to‐side difference in TLF thickness (2.39 ± 0.09 mm injection site vs. 2.36 ± 0.06 mm control site; mean ± SEM; Fig. 5) supporting the bilateral symmetry of TLF in the study sample. The high intraclass correlation of the measurements (*r* = 0.8150; *p* < 0.0001) revealing the consistency of ultrasound assessment across sides. Descriptive statistics for all main study variables, including TLF thickness, BMI, and peak pain intensity, are summarized in Table 1.

**Table 1:** Descriptive statistics of the main study variables. Values are presented as mean ± SEM and range for thoracolumbar fascia (TLF) thickness at the injection and contralateral control sites, body mass index (BMI), and peak pain intensity measured using a numerical rating scale (NRS)

	**Mean ± SEM**	**Range**
TLF thickness (injection site) (mm)	2.39 ± 0.09	1.50—2.91
TLF thickness (control site) (mm)	2.36 ± 0.06	1.83—2.88
BMI (kg/m^2^)	21.99 ± 0.37	18.99—24.86
Peak pain intensity (NRS 0–100)	60.00 ± 4.27	16—96

## Discussion

The present investigation demonstrates that ultrasound‐measured thoracolumbar fascia (TLF) thickness of the lower back in healthy volunteers is positively associated with both evoked deep‐tissue pain and body–mass index (BMI), while exhibiting bilateral symmetry. These findings enrich our understanding of fascia as an active participant in nociception and suggest novel avenues for research and clinical practice.

Fascia Thickness and Pain Sensitivity.

The thoracolumbar fascia thickness observed in our healthy cohort is consistent with previously reported ranges of ~ 1.0 to 4.6 mm in healthy adults [15], supporting the validity of our ultrasound measurements. Importantly, although our cohort was asymptomatic, greater fascial thickness within this non-pathological range was still associated with increased experimentally induced pain sensitivity. This observation may complement previous findings in chronic low back pain populations, where altered fascial morphology and reduced fascial mobility have been linked to pain-related conditions, suggesting that fascial structural characteristics may be associated with nociceptive sensitivity across both healthy and symptomatic individuals. Furthermore, our finding of a moderate correlation between TLF thickness and injection induced peak pain intensity underscores the structural possible contribution of fascia to experimentally induced deep‐tissue pain. In addition, its bilateral symmetry confirms the contralateral site as a reliable internal control in unilateral fascial interventions.

Heightened ongoing pain associated with thicker fascia in the lower back [6] can be attributed to several interrelated factors, including nociceptive innervation, mechanical properties, and neurophysiological changes. Histological evidence of abundant free nerve endings in the TLF [22] supports the notion that thicker connective tissue may harbor more nociceptive fibers or alter mechanical strain distribution under noxious stimuli. Moreover, Pirri and colleagues recently reported that chronic low back pain (CLBP) patients exhibit homogeneous thickening and loss of anisotropy within the TLF [15]. Increased fascial thickness may thus compromise the mechanical properties of the lower back over time by limiting gliding and adaptability [15] which may further amplify nociceptor strain during movement. Experimental stimulation of the human fascia has demonstrated that it can evoke pain responses [11, 16, 18, 25]. Furthermore, afferent signals from fascial tissue, known to induce long-term potentiation-like changes in pain sensitivity [17], may modulate pain perception during the onset and development of ongoing chronic conditions. Although fascia research reported correlations between fascial thickness and existing, ongoing pain conditions, it has remained uncertain whether fascial afferents at specific sites of the thickness assessments are directly involved in pain generation. Thus, the present study provides an experimental approach linking targeted afferent stimulation of the thoracolumbar fascia to pain outcomes that vary as a function of fascial thickness. In this study with healthy volunteers, the relationship between fascial structures and chemically induced pain links the individual differences in fascial morphology with individual pain sensitivity even before any injury or symptoms. Thus, this baseline fascial profile may serve as a potential biomarker for identifying individuals at increased risk of heightened pain intensity in occupational or athletic populations. While a positive correlation between thoracolumbar fascia thickness and induced pain intensity was observed in this study, results revealed that fascia thickness alone only partially accounts for evoked deep tissue pain perception. Approximately 37% of the inter-individual variability in induced peak pain is associated with thoracolumbar fascia thickness. Thus, as an outlook, other contributing factors such as peripheral or central sensitization or psychological influences are likely to play a significant role. For example, in patients with hypermobile Ehlers-Danlos syndrome, increased fascial thickness has been documented. However, the relationship with pain remains complex and might be associated with alterations in gliding properties leading to pain, joint instability, and dysfunction [26].

These findings underscore the multifactorial nature of pain processing, where structural changes in connective tissue represent only one aspect within a broader biopsychosocial framework. Notably, the association between thoracolumbar fascia thickness and pain intensity remained significant after controlling for BMI, although with a moderate reduction in correlation strength. This finding suggests that the observed relationship is not fully explained by body mass alone and may reflect specific structural characteristics of the fascia associated with nociceptive sensitivity. Potential contributors may include local differences in extracellular matrix organization, fascial afferent fiber architecture, or afferent fiber innervation density. At the same time, the attenuation of the correlation after BMI adjustment indicates that metabolic or obesity-related factors may still contribute to the observed associations. Nonetheless, given the exploratory nature and limited sample size, the findings should be interpreted as preliminary evidence requiring confirmation in larger, adequately powered studies.

### Body–mass index and fascial morphology

The correlation between fascia thickness and BMI is a topic of interest in various medical fields. The present study identified a moderate positive association between BMI and fascia thickness of the lower back. Results from Wilke and colleagues support the relation of BMI and lower back fascia thickness of healthy volunteers [28]. Further research on total hip arthroplasty patients underlines this at surgical sites [1]. This relationship is important in surgical contexts, where soft tissue thickness can influence perioperative factors and outcomes. In the context of spine surgery, BMI showed a moderate correlation with posterior subcutaneous fat thickness [14] suggesting a relationship between BMI and localized fat distribution in these anatomical areas. The well‐established epidemiological link between obesity and low back pain prevalence [19] may thus be partly mediated by obesity‐related changes in fascial thickness. Given that BMI does not distinguish between lean and fat mass, future studies should employ direct body‐composition assessments and consider physical activity levels to delineate the distinct contributions of adipose versus muscle tissue to fascial architecture. In contrast, Pirri and colleagues reported no correlation between the thickness of the thoracolumbar fascia in patients with chronic lower back pain and individual BMI but suggested to be associated with altered fascial remodeling [15]. This indicates that factors other than BMI might influence fascia thickness in certain conditions. Nonetheless, together with the pain correlation, this relationship between BMI, fascia thickness and induced pain intensity supports the multifactorial determinants of fascial biomechanics and pain sensitivity in healthy individuals. Beyond thickness, recent in vivo elastography studies highlight the importance of fascial mechanical properties in pain states. Tomita and colleagues used ultrasound shear‐strain imaging and demonstrated elevated cumulative and maximum shear strain and strong correlations with pain and disability scores in nonspecific low back pain patients despite similar TLF thickness [23]. Furthermore, Brandl and colleagues quantified TLF deformation during standardized trunk extension and found reduced fascial deformability in acute LBP patients, implicating increased stiffness in impaired load distribution and nociception [2, 3]. Collectively, these studies suggest that fascial stiffness and strain, alongside thickness, shape nociceptor activation under mechanical or chemical stimulation, inviting more comprehensive in vivo assessments of fascial biomechanics. In addition, the observed positive association between body mass index and pain sensitivity, accounting for approximately one third of the inter-individual variability, emphasizes the potential relevance of systemic factors in modulating nociceptive responses and highlights further the body composition as a potential contributor to individual pain sensitivity. Nonetheless, the observed association between BMI and pain sensitivity is unlikely to be explained by body mass alone but may reflect underlying metabolic and inflammatory processes. Adipose tissue functions as an active endocrine organ, releasing adipokines such as leptin and adiponectin, which have been shown to modulate inflammatory pathways and pain perception [9, 13]. In addition, elevated BMI is associated with low-grade systemic inflammation, as reflected by increased levels of C-reactive protein, which may contribute to altered connective tissue properties and nociceptive sensitivity [24]. These mechanisms may also influence fascial remodeling, including changes in extracellular matrix composition and tissue stiffness [10]. Together, this suggests that the relationship between BMI, TLF structure, and pain sensitivity may be mediated by complex biochemical and inflammatory pathways rather than purely mechanical factors.

### Clinical and research perspectives

Our data support the concept of TLF thickness or BMI as a putative biomarker for pain sensitivity although integration of mechanical‐property data (e.g., shear strain or deformability) may enhance predictive power. Clinically, ultrasound‐based screening of fascial thickness with stiffness could identify individuals at elevated risk for CLBP, guiding early preventive interventions such as targeted myofascial release or therapeutic exercise. Since the reported somatosensory crosstalk between superficial and deep tissues [11], combining quantitative sensory testing as a measurement of the tenderness of the skin with ultrasound elastography and histological analyses [20] would elucidate the multiscale mechanisms linking fascial structure and nociception.

### Limitations

The modest sample size (n = 20) may constrict the precision of correlation estimates and limit subgroup analyses. And, volunteers were predominantly young and pain‐free, restricting generalizability to clinical populations. Furthermore, BMI as a surrogate for obesity fails to capture fat distribution or muscle mass. Thus, advanced body‐composition tools are necessary in future work. Furthermore, a formal mediation analysis may provide additional insight into the relationships between BMI, thoracolumbar fascia thickness, and pain intensity. However, such approaches typically require larger sample sizes to ensure reliable estimation of indirect effects.

## Conclusions

This study demonstrates that in healthy adults, increased thoracolumbar fascia (TLF) thickness as measured by ultrasound is positively correlated with both heightened pain intensity during experimentally induced deep tissue stimulation and higher body mass index (BMI), even in the absence of pathology. Additionally, TLF thickness showed bilateral symmetry and high measurement reliability, supporting its suitability for intra-individual comparisons and routine imaging protocols. These findings may be clinically relevant, as the TLF thickness may not only serve as a structural marker but also offer predictive value for pain vulnerability under mechanical stress or overload. The association with BMI further supports the potential interaction between metabolic status and fascial adaptation. Together, these insights support the role of ultrasound-based fascia assessment as a practical, non-invasive tool for early risk stratification, targeted interventions in musculoskeletal pain management, and monitoring the musculoskeletal health impacts of weight-related interventions. Moreover, its accessibility and reproducibility suggest potential utility as a preventive screening tool to identify individuals who may benefit from proactive care.

## Data Availability

The datasets used and/or analyzed during the current study are available from the corresponding author on reasonable request.

## Abbreviations

TLF: Thoracolumbar fascia
BMI: Body mass index
NaCl: Sodium chloride
NRS: Numerical rating scale
CLBP: Chronic low back pain
SD: Standard deviation
µL: Microliter
kg: Kilogram
m2: Square meter
ICC: Intraclass correlation coefficient
SEM: Standard error of the mean
